# Les urgences gynéco-obstétricales au service de gynécologie obstétrique de Sousse: étude épidémiologique et devenir des consultants

**DOI:** 10.11604/pamj.2022.43.53.32867

**Published:** 2022-10-03

**Authors:** Imen Bannour, Manel Limam, Ghada Rjiba, Rania Bannour, Thouraya Ajmi

**Affiliations:** 1 Université de Sousse, Faculté de Médecine de Sousse, 4000, Sousse, Tunisie,; 2 Hôpital Farhat Hached, Service de Gynécologie Obstétrique, « Laboratoire de Recherche “LR12ES03” », 4000, Sousse, Tunisie,; 3 Département de Médecine de Famille et de Médecine Communautaire, «Laboratoire de Recherche “LR12ES03”», 4002 Sousse, Tunisia

**Keywords:** Urgences, gynéco-obstétrique, motifs de consultation, diagnostics, Emergencies, obstetrics and gynecology, reasons for consultation, diagnostic

## Abstract

**Introduction:**

l'objectif de ce travail était d'établir le profil clinique des patientes consultant aux urgences gynéco-obstétricales et relever les motifs de consultation et le devenir des patientes.

**Méthodes:**

une étude d'observation descriptive rétrospective uni-centrique a été menée. Ont été incluses les patientes ayant consulté entre le 1^er^ janvier et le 31 décembre 2018. N'ont pas été incluses les urgences obstétricales après 36 semaines d'aménorrhée. On a tiré au sort 4 mois de l'année 2018, soit un mois par saison. Puis on a tiré au sort 2 semaines pour chaque mois. Une fiche de collecte de données a été élaborée pour les fins de ce travail.

**Résultats:**

au total, 2007 patientes ont été incluses dans notre étude parmi 15553 consultantes aux urgences gynécologiques durant l'année 2018. Nous avons constaté que le nombre le plus important de consultantes a été enregistré en début de semaine et entre 7h et 19h. Les motifs obstétricaux de consultation les plus fréquemment observés étaient: la douleur pelvienne (39,6%), les métrorragies (23,8%) et les vomissements (8,7%). Les motifs gynécologiques de consultation les plus fréquemment rapportés étaient: la douleur pelvienne (54,2%), puis la métrorragie (18,8%) et la mastodynie (7,1%). Parmi les participantes à l'étude 66,82% ont bénéficié d'une échographie, 23% ont bénéficié d'un dosage de la beta HCG. Le devenir le plus fréquent des patientes consultantes aux urgences gynécologique et obstétricales était le retour à domicile.

**Conclusion:**

la majorité des patientes consultant aux urgences ne présentent pas de pathologies relevant de l'urgence.

## Abstract (en)

**Introduction:**

the objective of this work was to establish the clinical profile of patients consulting in gyneco-obstetrical emergencies and identify the reasons for consultation and the becoming of the consultants.

**Methods:**

a one-center retrospective descriptive observational study was performed including patients who consulted between January 1^st^ and December 31^st^, 2018. Obstetric emergencies after 36 weeks of amenorrhea were not included. We drew lots 4 months of the year 2018 (one month per season). Then we drew lots 2 weeks of each month. A data collection sheet was developed for the purposes of this work.

**Results:**

a total of 2007 patients were included in our study among 15,553 gynecological emergency room consultants during 2018. We found that the largest number of consultants was recorded at the start of the week and between 7 am and 7 pm. The most frequently obstetric reasons observed for consultation were pelvic pain (39.6%), bleeding (23.8%) and vomiting (8.7%). The most frequently gynecological reasons for consultation were pelvic pain (54.2%), then metrorrhagia (18.8%) and mastodynia (7.1%). Of the study participants, 66.82% received an ultrasound, 23% received a beta HCG test. The majority of emergency room consultants were referred to their home.

**Conclusion:**

the majority of patients visiting the emergency room do not have any emergency-related pathologies.

## Introduction

Les urgences de gynécologie obstétrique sont toujours encombrées par des malades qui ne relèvent pas de l'urgence réelle. Cet encombrement est à l'origine d'un retard de prise en charge surtout dans les pays en voie de développement qui souffrent d'un manque des ressources humaines (médecins, infirmiers, ouvriers…) et matérielles. Donc les soignants qui doivent gérer en même temps la salle de naissance et les urgences au cours de la garde sont toujours débordés par des fausses urgences [1]. Plusieurs auteurs ont cherché des méthodes qui permettraient d'établir un ordre de priorité des urgences et d'autres ont essayé de mettre en place un premier relais médical avant les consultations d'urgence [2].

La gestion des urgences reste ainsi une difficulté pour grand nombre de services surtout dans les pays pauvres, par la surcharge de travail qu'elles engendrent ainsi que pour la difficulté du tri des pathologies. Afin de contribuer à cette réflexion, bien étudier le type de pathologies rencontrées parmi nos urgences gynécologiques, leur degré de sévérité et leur évolution, améliorer la prise en charge et la régulation aux urgences et cibler la formation des médecins, nous avons décidé de mener ce travail ayant pour objectif d'établir le profil épidémiologique et clinique des patientes consultant aux urgences gynéco-obstétricales du Centre de maternité et de néonatologie du CHU Farhat Hached de Sousse durant l'année 2018 et relever les motifs de consultation, les pathologies rencontrées et le devenir des patientes.

## Méthodes

**Cadre de l'étude:** le service de gynécologie obstétrique du CHU Farhat Hached de Sousse est une des plus grandes maternités du centre Tunisien, c'est une maternité de niveau III qui a réalisé en 2018, 9590 accouchements. Son activité concerne les pathologies pelviennes gynécologiques bénignes et cancéreuses, la sénologie, il existe aussi une activité de diagnostic anténatal, de procréation médicalement assistée, de chirurgie gynécologique cancérologique et fonctionnelle.

Les données du service, obtenues à partir des statistiques de chaque fin d'année montrent qu'au cours de l'année de notre étude les urgences gynécologiques ont accueilli 15799 consultantes et que les urgences obstétricales ont accueilli 10699 patientes tandis que la salle d'enregistrement RCF a accueilli 5662 femmes soit un total de 32 160 patientes durant l'année 2018.

L'unité des urgences gynécologiques et obstétricales fonctionne tous les jours 24 heures sur 24 et elle est située au rez de chaussé de l'établissement. Cette unité comprend, une salle d'attente, une salle d'examen pour les urgences gynécologiques et obstétricales (< à 36 SA), une salle d'examen pour les urgences obstétricales (> à 36 SA), une salle d'échographie, une salle comportant 3 lits ou se font les enregistrements du RCF.

En effet, les internes ont la tâche d'examiner les patientes, prendre les avis et de rédiger les admissions alors que le résident doit réaliser l'examen pour toutes les patientes aux urgences gynécologiques et aux patientes qui posent problème aux urgences obstétricales et réaliser les échographies pour les patientes qui consultent aux urgences gynécologiques et obstétricales.

Une infirmière est chargée de l'accueil des patientes venant consulter aux urgences gynécologiques et obstétricales ainsi que celles venues pour faire un enregistrement du RCF, elle est aussi chargée des prélèvements et de prendre des notes dans le registre de l'accueil (heure d'arrivée des patientes, motifs de consultation, âge et provenance).

L'urgence obstétricale et la salle d'enregistrement du RCF sont gérées par deux sages femme et un interne de 8H à 13 H, assistés par le résident des urgences et le sénior de garde en cas de besoin puis par une seule sage-femme durant la garde soit de 13H à 8H du lendemain, assistée par le résident et le sénior de garde en cas de besoin. Les échographies réalisées aux urgences sont réalisées par le résident de garde aux urgences assisté par le sénior de garde en cas de besoin.

Le service des urgences prend en charge toute patiente se présentant pour un éventuel problème gynéco-obstétrical urgent. Il existe différents modes d'admission dans ce service (les patientes viennent d'elles même en consultation, adressées par un médecin, par le service des urgences de médecine générale ou par un service d'hospitalisation du CHU, transférées d'une autre structure de soins et parfois elles sont convoquées par le service des urgences gynécologiques lui-même.

Dans ce service d'urgence, la patiente est accueillie par un interne qui prend ses coordonnées, le motif de consultation, vérifie le degré d'urgence, puis installe la patiente en salle d'examen et prend ses constantes vitales. Les patientes sont vues par ordre d'arrivée et/ou selon le degré de gravité supposé, évalué par l'infirmière à l'accueil. La patiente est ensuite vue par le résident de garde qui l'examine et demande certains examens complémentaires (biologie, échographie) s'il le juge nécessaire. Il peut faire appel si besoin au sénior de garde. L'observation médicale est rédigée sur le registre des urgences et en cas d'hospitalisation l'observation est rédigée sur un dossier d'observation.

Il existe différents modes de sortie de ce service (retour à domicile définitif, retour à domicile avec prescription d'un contrôle biologique et/ou échographique aux urgences, retour à domicile avec traitement chirurgical prévu ultérieurement, nouvelle convocation en consultation dans la consultation externe, transfert vers un autre service, hospitalisation dans le service).

**Type de l'étude:** nous avons mené une étude d'observation descriptive rétrospective uni-centrique sur les registres d'activités des consultations au service des urgences gynécologiques du Centre Hospitalier Universitaire (CHU) Farhat Hached de Sousse entre le 1^er^ janvier 2018 et le 31 décembre 2018.

**Participants à l'étude et recrutement des échantillons:** ont été incluses les patientes ayant consulté ce service pendant cette période. N'ont pas été incluses les patientes qui ont consultées les urgences obstétricales après 36 semaines d'aménorrhée (36SA), ainsi que les réquisitions médicales. Nous avons tiré au sort 4 mois de l'année 2018, soit un mois par saison (janvier pour l'hiver, mars pour le printemps, juillet pour l'été et octobre pour l'automne). Puis on a tiré au sort 2 semaines pour chaque mois soit 14 jours pour chaque mois. Toutes les consultations répondant à nos critères de sélection durant ces périodes ont été prises en considération.

**Conception de l'étude:** l'urgence médicale était définie comme un état clinique stable ou pouvant s'aggraver nécessitant l'hospitalisation ou engageant le pronostic vital, requérant une prise en charge rapide (2). Pour les diagnostics retenus nous nous sommes basé sur la classification internationale des maladies dans sa dixième version (CIM 10) étant donné que la dernière version (CIM 11) n'est disponible qu'en anglais. Pour le recueil des données, une fiche de collecte de données a été élaborée pour les fins de ce travail, inspirée d'une revue de la littérature. Le recueil des données s'est fait en consultant le registre des admissions des urgences gynéco-obstétricales de l'année en question et le registre d'inscription tenue par l'infirmière d'accueil de ces urgences.

**Analyse des données:** la saisie et l'analyse des données ont été effectuées par le logiciel EPI info version 2020. La normalité des variables a été testée par le test non paramétrique de Kolmogorov Smirnov. Les variables quantitatives qui ont une distribution gaussienne, ont été exprimées en moyenne et écart type alors que pour celles dont la distribution n'était pas normale on a utilisé la médiane avec les valeurs extrêmes. Les variables qualitatives, ont été exprimées en fréquences absolues (effectifs) et fréquences relatives (pourcentages).

## Résultats

Au total, 2007 patientes ont été incluses dans notre étude parmi 15553 consultantes aux urgences gynécologiques durant l'année 2018. La répartition des patientes est comme suit, en janvier 363 patientes (18,09%), en mars 577 patientes (28,75%), en juillet 522 (26,01%), en octobre 545 (27,15%).

Nous avons constaté que le nombre le plus important de consultantes a été enregistré en début de semaine: lundi (n= 359; 17,88%), Mardi (n= 3>27; 16,29%), mercredi (n= 339; 16,89%), jeudi (n=274; 13,65%), vendredi (n=294; 14,64%), samedi (n= 211; 10,51), dimanche (n=203; 10,11%).

Nous avons constaté que la majorité des patientes consultent entre 7h et 19h; soit 31,95% (n=285) entre 7h et 13h, 31,05% (n=277) entre 13 et 19h alors que 24,44% (n=218) entre 19h et 00h00 et 12,56% (n=112) entre 00h00 et 7h. Les patientes adressées représentent uniquement 6,53% (n=131) de la population d'étude alors que 93,46% (n=1872) sont venues par leurs propre initiative.

L'âge moyen des patientes était de 32,36 +/- 12,29 avec des extrêmes allant de 4 ans à 86 ans. La majorité de nos patientes étaient des femmes mariées (n= 1817; 93,08%) alors que seulement 6,76% étaient célibataires (n=132). Les patientes résidaient dans 84,33% des cas (n=1526) au gouvernorat de Sousse. La majorité des femmes consultantes sont des multipares (60%; n=1049). La médiane de parité était de 5,5 avec les extrêmes allant de 1 à 11.

Au cours de notre étude 1106 patientes ont consulté pour des motifs obstétricaux et 789 ont consulté pour des motifs gynécologiques. Les motifs obstétricaux de consultation les plus observés dans cette étude étaient: la douleur pelvienne 438 cas (39,6%), les métrorragies 263 cas (23,8%), les vomissements 96 cas (8,7%). Les motifs gynécologiques de consultation rapportés étaient: la douleur pelvienne 428 cas (54,2%), la métrorragie 148 cas (18,8%), la mastodynie 56 cas (7,1%).

Parmi les participantes à l'étude 66,82% (n=1339) ont bénéficié d'une échographie, 23% (n=461) ont bénéficié d'un dosage de la beta HCG, 6,73% (n=136) ont bénéficiées d'un enregistrement du rythme cardiaque fœtal (RCF) et 86 ont bénéficiées d'autres examens biologiques. Les diagnostics les plus fréquemment retrouvés chez nos patientes qui n'étaient pas enceintes au moment de la consultation étaient les infections génitales basses (9,78%; n=67/706) suivie des infections génitales hautes (4.96%; n=34/706) (Tableau 1).

**Tableau 1:** les diagnostics retenus non associés à la grossesse (n=706)

Code CIM10	Diagnostics	Effectif (n)	Pourcentage (%)
	Diagnostique non établi adressée aux URG médicales	74	10,48
N77.1	infection génitale basse	67	9,49
N70	Infection génitale haute(IGH)	34	4,82
O86	infection post-op	33	4,67
N83.2	kyste de l´ovaire	33	4,67
N63	tuméfaction mammaire	29	4,11
N61	abcès du sein	21	2,97
O00	Grossesse extra-utérine GEU	21	2,97
N87	cancer du col	14	1,98
O03/O04	avortement	13	1,84
N83.0	ovulation hémorragique	12	1,70
N75.1	abcès de la glande de bartholin	10	1,42
N83.5	torsion d´annexes	10	1,42
N89.8	déchirure vaginale	9	1,27
N75.8	bartholinite	5	0,71
N71	endométrite	4	0,57
O21	vomissement gravidique	3	0,42
N75.0	kyste de la glande de bartholin	1	0,14
N83.9	kyste rompu	1	0,14
O01	mole hydatiforme	1	0,14
	autre	311	44,05

Les diagnostics les plus fréquemment retrouvés chez les patientes enceintes étaient: la menace d'avortement (222/1052 cas soit 21,72%), les menaces d'accouchement prématuré (152/1052 cas soit 14,97%), les avortements (115/1052 cas soit 11,15%) et les vomissements gravidiques (82/1052 cas soit 8,02%) (Tableau 2).

**Tableau 2:** les diagnostics retenus associés à la grossesse (n=1052)

Code CIM10	diagnostic	Effectif (n)	Pourcentage (%)
O20.0	menace d´avortement	222	31,44
O60	MAP	153	21,67
O03/O04	avortement	114	16,15
O21	vomissement gravidique	82	11,61
O14	pré éclampsie	58	8,22
O00	GEU	53	7,51
O23.0	PNA femme enceinte	49	6,94
N77.1	infection génitale basse	41	5,81
O42	RPM	28	3,97
O02.1	MFIU	25	3,54
P05	retard de croissance	7	0,99
N70	IGH	5	0,71
N83.2	kyste de l´ovaire	5	0,71
O44	placenta prævia saignant	5	0,71
	Diagnostique non posée adressée aux URG medicales	3	0,42
O45	suspicion HRP	3	0,42
O86	infection post-op	2	0,28
N75.1	abcès de la glande de bartholin	1	0,14
N61	abcès du sein	1	0,14
N83.9	kyste rompu	1	0,14
O01	mole hydatiforme	1	0,14
N83.0	ovulation hémorragique	1	0,14
	autre	192	27,20

Nous avons noté que le devenir le plus fréquent des patientes consultantes aux urgences gynécologiques était le retour à domicile et ceci est valable pour les patientes enceintes (71,33%; n=663) et non enceinte (72,51%; n=810). Pour les consultantes non enceintes, 769 (89,58%) patientes ont été adressées à leurs domicile, 82 patientes (9.26%) ont été hospitalisées (Tableau 3). Pour les consultations obstétricales, 853 patientes (76,35%) ont été adressées à leurs domicile et 210 patientes (18,84%) ont été hospitalisées (Tableau 4).

**Tableau 3:** devenir des patientes non enceintes (n=887)

Devenir	Nombre de consultantes	Pourcentage par rapport aux consultations gynécologiques (%)
Retour à domicile avec un traitement ambulatoire	769	89,58
Hôpital du jour	6	0,64
Hospitalisation pour surveillance	41	4,63
Intervention chirurgicale	41	4,63
Hospitalisation dans un autre service	4	0,45

**Tableau 4:** devenir des patientes enceintes (n=1117)

Devenir	Nombre de consultantes	Pourcentage par rapport aux consultations obstétricales (%)
Retour à domicile avec un traitement ambulatoire	853	76,35
Hôpital du jour	25	2.27
Hospitalisation pour surveillance	210	18.84
Intervention chirurgicale	25	2,27
Hospitalisation dans un autre service	3	0,26

## Discussion

Il existe très peu de données dans la littérature concernant la fréquence des urgences gynécologiques. Dans notre étude, le nombre de consultations était en moyenne de 1296 consultation par mois. Dans l'étude de Coutin AS et Vaucel E [3], les auteurs ont montré une difficulté en gynécologie la nuit et le week-end car les urgences étaient le plus fréquentées pendant ces périodes. Dans notre étude nous avons pu observer que la majorité des consultations étaient en début de semaine. Dans notre étude nous avons trouvé également que les consultations étaient plus fréquentes (63%; n=562) entre 7h et 19h. Un très grand nombre de patientes se présentent aux urgences gynécologiques et obstétricales sans avoir été adressées par un professionnel de santé [4]. Dale *et al*. retrouvent que 90,1% des patientes consultent aux urgences sans avoir été adressées par un médecin généraliste et que seulement 0,5% de ces patientes sont hospitalisées par contre 29,5% des patientes adressées par un médecin généraliste sont hospitalisées [5]. Dans l'étude d'Alouini *et al*. la majorité des patientes se présentaient de leur propre initiative [1].

Dans notre étude nous avons retrouvé qu'uniquement 6,53% (n=131) de nos patientes ont été référées et que 93,47% (n=1872) des patientes sont venues par leur propre initiative. Concernant les caractéristiques des patientes, l'âge moyen des patientes est de 30 ans en moyenne [4]. Dans notre étude l'âge moyen des patientes consultant aux urgences gynécologiques était de 32,36 ans. Selon les auteurs, les métrorragies du premier trimestre de la grossesse et les douleurs pelviennes [6] représentent les motifs les plus fréquemment rencontrées dans les urgences en gynécologies. Dans notre étude, les motifs de consultation gynécologiques les plus fréquents étaient les douleurs pelviennes avec 54,2%, suivies des métrorragies avec 18,8% puis les mastodynies (7,1%). De nombreuses patientes consultent en urgence en début de grossesse, l'objectif de la consultation est souvent de confirmer la grossesse, d'obtenir une échographie ou de vérifier le bon déroulement de la grossesse. Les douleurs abdomino-pelviennes constituent le motif de consultation le plus fréquent. La difficulté rencontrée par les soignants est de distinguer la douleur organique des symptômes physiologiques inhérents à la grossesse sans avoir à réaliser d'examens para cliniques [4].

Dans notre étude, les motifs de consultation obstétricaux les plus fréquemment retrouvés étaient la douleur pelvienne avec 38%, les métrorragies avec 23,8% et les vomissements avec 8,7%. L'examen complémentaire le plus pratiqué dans les urgences gynécologiques était l'échographie pelvienne, ce qui peut se comprendre vu les symptomatologies mises en avant par les patientes (douleurs pelvienne et métrorragies). D'autres auteurs ont montré l'intérêt et l'apport indispensable de l'échographie dans le diagnostic des GEU et des pathologies utéro-ovariennes [7]. Dans la littérature, L'examen complémentaire le plus pratiqué était l'échographie pelvienne [1]. Dans notre étude 1339 patiente soit 66,22% ont bénéficié d'une échographie. Pour Nadel *et al*. [8], le dosage des béta HCG reste un examen essentiel associé à l'échographie pour exclure les grossesses ectopiques. Dans notre étude ¼ des patientes ont bénéficiées d'un dosage de béta HCG (n=461; 22.8%).

Au cours de notre étude, nous avons noté que les diagnostics les plus retrouvés chez les femmes non enceintes étaient les infections génitales basses et les infections génitales hautes alors que les diagnostics les plus fréquents chez les femmes enceintes étaient la menace d'avortement prématuré et la menace d'accouchement prématuré. Conformément à nos résultats, Curtis *et al*. trouvent que près de la moitié des consultations aux urgences gynécologiques sont dues à des infections des voies génitales [9]. Dans la littérature, 80% des patientes ont pu rentrer à domicile à l'issue de la consultation [1,5]. Dans notre étude, nous avons pu constater que le devenir le plus fréquent des patientes consultantes aux urgences gynécologique était le retour à domicile et ceci est valable pour les patientes enceintes (71,33%; n=663) et non enceinte (72,07%; n=805).

Dans une étude de Alouini *et al*., on a pu montrer que la majorité des consultations aux urgences gynéco-obstétricales n'ont aucun caractère d'urgence et sont principalement représentées par des pathologies bénignes voire une absence totale de pathologie [1]. La grossesse débutante était la raison principale du recours aux urgences gynécologiques et obstétricales [1]. On a noté dans notre étude que les 5 diagnostics les plus fréquents étaient: les menaces d'avortement, les menaces d'accouchement prématuré, les avortements, les vomissements gravidiques et les infections génitales basses.

En effet, on a pu observer que la majorité des motifs de consultations relèvent de la médecine en ville. Même s'il est très délicat de quantifier le nombre de consultations qui auraient pu être gérées en ville vu que les symptômes sont peu spécifiques et évocateurs de pathologies potentiellement grave. Il est peut-être plus facile de quantifier le nombre d'urgences vraies à partir du nombre de patientes hospitalisées. En effet on peut considérer que toute patiente hospitalisée était une urgence médicale ou chirurgicale. Dans notre étude 9,26% des patientes non enceintes ont été hospitalisées et 21,37% des patientes consultantes pour des motifs obstétricaux ont été hospitalisées. Ces consultations non urgentes, encombrent les urgences, entrainent une exhaustion et un épuisement du personnel médical et paramédical et retardent la prise en charge des vraies urgences.

Nous pouvons dégager les axes d'amélioration suivants pour améliorer la prise en charge des patientes consultant aux urgences gynécologiques et obstétriques: il serait intéressant d'étudier en amont des structures d'urgence le type de population et son niveau socio-économique qui fréquente les urgences gynécologiques et obstétriques afin de cibler cette population; il serait aussi intéressant d'étudier la répartition hebdomadaire et journalière des venues non programmées aux urgences gynécologiques et obstétriques afin de déterminer les jours les plus affluents et les pics horaires de fréquentation afin d'optimiser le fonctionnement des urgences.

Mettre en place un système de triage afin de déterminer avec quelle rapidité la patiente devrait être prise en charge. Ce système a été développé et appliqué à la médecine civile hospitalière notamment par les anglo-saxons au cours des années 70. Il comprend plusieurs niveaux de gravité et prend en compte différents critères [10]. Il permet d'optimiser la prise en charge des usagers et de réduire les délais d'attente. Il est maintenant utilisé dans de nombreux pays tels que le Canada [10], les États-Unis [7,11], l'Australie [12] ou le Royaume-Unis [13] pour favoriser et améliorer la coopération entre l'hôpital et le secteur libéral. En effet, orienter la patiente vers son médecin généraliste, son gynécologue, sa sage-femme ou son obstétricien, quand cela est possible, réduirait le nombre de consultations aux urgences. D'un point de vu de la démographie médicale.

**Limites de l'étude:** notre étude présente certaines limites qu'Il s'agit d'une étude rétrospective mono centrique, Nous avons exclu les patientes dont le terme de la grossesse était supérieur à 36 SA. Cela ne nous a pas permis d'étudier les urgences du post-partum. Le caractère rétrospectif du recueil de données a aussi limité le nombre d'informations inscrites dans les dossiers entre autres les données sociodémographiques et économiques des patientes. Nous n'avons pas distingué entre les patientes dont la grossesse était confirmée avant la consultation aux urgences et la grossesse qui est découverte la première fois lors de la consultation aux urgences gynécologiques.

La force de notre étude réside dans le fait que c'est la première étude qui donne un état des lieux de nos urgences gynécologiques et obstétriques en Tunisie.

## Conclusion

Les urgences de gynécologie obstétrique sont toujours encombrées par des malades qui ne relèvent pas de l'urgence réelle. Cet encombrement entraine une difficulté de prise en charge surtout dans les pays pauvres qui souffrent d'un manque des ressources humaines et matérielles. Nous avons décrit le profil épidémiologique et clinique des patientes consultant aux urgences gynéco-obstétricales du Centre de maternité et de néonatologie du CHU Farhat Hached de Sousse en 2018 ainsi que les principaux motifs de consultation et les pathologies rencontrées et le devenir des patients. Nous avons relevé que la grande majorité des consultantes aux urgences ne relèvent pas de l'urgence réelle.

### Etat des connaissances sur le sujet

- Les urgences de gynécologie obstétrique sont toujours encombrées par des malades qui ne relèvent pas de l'urgence réelle;
- Cet encombrement est à l'origine d'un retard de prise en charge surtout dans les pays en voie de développement qui souffrent d'un manque des ressources humaines (médecins, infirmiers, ouvriers…) et matérielles.

### Contribution de notre étude à la connaissance

- Établir le profil épidémiologique et clinique des patientes consultant aux urgences gynéco-obstétricales d'un centre tunisien de maternité et de néonatologie de niveau III;
- Relever les motifs de consultation, les pathologies rencontrées et le devenir des patientes consultant au urgences de gynécologie obstétrique.
